# Analysis of the Larissa Heart Failure Risk Score: Predictive Value in 9207 Patients Hospitalized for Heart Failure from a Single Center

**DOI:** 10.3390/jpm13121721

**Published:** 2023-12-17

**Authors:** Andrew Xanthopoulos, John Skoularigis, Alexandros Briasoulis, Dimitrios E. Magouliotis, Alex Zajichek, Alex Milinovich, Michael W. Kattan, Filippos Triposkiadis, Randall C. Starling

**Affiliations:** 1 Department of Cardiology, University General Hospital of Larissa, 41110 Larissa, Greece; iskoular@uth.gr (J.S.);; 2 Department of Clinical Therapeutics, Faculty of Medicine, Alexandra Hospital, National and Kapodistrian University of Athens, 11528 Athens, Greece; abriasoulis@med.uoa.gr; 3 Unit of Quality Improvement, Department of Cardiothoracic Surgery, University of Thessaly, 41110 Larissa, Greece; dimitrios.magouliotis.18@ucl.ac.uk; 4 Department of Quantitative Health Sciences, Cleveland Clinic, Cleveland, OH 44196, USAkattanm@ccf.org (M.W.K.); 5 Department of Cardiovascular Medicine, Heart and Vascular Institute, Kaufman Center for Heart Failure, Cleveland Clinic, Cleveland, OH 44195, USA

**Keywords:** Larissa heart failure risk score, stratification, heart failure, hospitalized, mortality

## Abstract

Early risk stratification is of outmost clinical importance in hospitalized patients with heart failure (HHF). We examined the predictive value of the Larissa Heart Failure Risk Score (LHFRS) in a large population of HHF patients from the Cleveland Clinic. A total of 13,309 admissions for heart failure (HF) from 9207 unique patients were extracted from the Cleveland Clinic’s electronic health record system. For each admission, components of the 3-variable simple LHFRS were obtained, including hypertension history, myocardial infarction history, and red blood cell distribution width (RDW) ≥ 15%. The primary outcome was a HF readmission and/or all-cause mortality at one year, and the secondary outcome was all-cause mortality at one year of discharge. For both outcomes, all variables were statistically significant, and the Kaplan–Meier curves were well-separated and in a consistent order (Log-rank test *p*-value < 0.001). Higher LHFRS values were found to be strongly related to patients experiencing an event, showing a clear association of LHFRS with this study outcomes. The bootstrapped-validated area under the curve (AUC) for the logistic regression model for each outcome revealed a C-index of 0.64 both for the primary and secondary outcomes, respectively. LHFRS is a simple risk model and can be utilized as a basis for risk stratification in patients hospitalized for HF.

## 1. Introduction

Heart failure (HF) is a pandemic characterized by high prevalence and incidence [1]. At least 15 million patients in Europe suffer from HF, according to the European Society of Cardiology (ESC) [2]. In the United States, data from the ARIC Community Surveillance (2005–2013) revealed a significant number of annual first episodes of acute decompensated HF in the range of 25.7–34.8 per 1.000 person years for patients aged ≥ 75 years [3]. Although a number of life-saving therapeutic strategies have been implemented in the last two decades, both pharmaceutical (ACE-inhibitors, ARB, ARNI, beta-blockers, mineralocorticoid antagonists) and interventional (primary percutaneous coronary intervention, cardiac resynchronization therapy, implantable cardioverter-defibrillators, and left ventricular assist devices), recent data from the ESC-HFA Heart Failure Long-Term Registry (ESC-HF-LT) revealed 1-year all-cause mortality rates of 23.6% for hospitalized HF (HHF) and 6.4% for chronic HF (CHF) [4].

Timely and precise risk stratification of HF patients is of outmost importance, especially in those acutely hospitalized, where the clinical decision is often challenging [5]. Consequently, several predictive scores have been evolved and validated in HHF [6,7,8,9]. Nonetheless, in the majority of these prognostic models, numerous parameters (multivariable risk scores) are incorporated, making their calculation difficult and frequently requiring the utilization of sophisticated calculators [10]. Accordingly, we validated the predictive value of the very simply calculated Larissa Heart Failure Risk Score (LHFRS) [11,12] in a large population of patients hospitalized for HF from a single health system.

## 2. Materials and Methods

### 2.1. Patients

A total of 13,309 hospital admissions for HF, corresponding to 9207 patients, were extracted from the Cleveland Clinic Health System electronic health record system (HER), which satisfied the inclusion criteria:

(1) Occurrence in Ohio (from December 2009 through May 2015) with HF as the primary reason for hospital admission.
(2) Admission hemoglobin value ≥ 10 g/dL.
(3) Admission red blood cell distribution width (RDW) determination.
(4) Presence of data on hypertension (HTN) history and myocardial infarction (MI) history.

### 2.2. The Larissa Heart Failure Risk Score

The derivation of LHFRS has been previously described in detail. [11] Variables included are HTN history, MI history, and RDW at admission. Absence of HTN history, presence of MI history, and admission RDW value ≥15% are assigned 2, 1, and 1 points, respectively. Patients with LHFRS = 0 have the best score, whereas those with LHFRS = 4 have the worst score.

### 2.3. Definitions

HTN history was defined as having a diagnosis of HTN on the problem list during admission; MI history was defined as having an MI diagnosis any time prior to admission; and RDW values that were measured on the day of admission were used. Regarding all-cause mortality, the date of death was used. This consisted of either: (a) the date of death if the patient died in the hospital and thus it was recorded; or (b) the date of death according to the Ohio Death Index, which is the state’s record of people who died in Ohio or were Ohio residents and died outside of Ohio. The ICD codes used for the present analysis are depicted in Table 1.

### 2.4. Outcomes 

The primary outcome of the present analysis was either a HF readmission or all-cause mortality within 1-year of discharge. The secondary outcome was all-cause mortality within 1-year of discharge. The first occurrence of readmission or death after discharge was taken to be the event time. Patients were followed through November 2017, though censoring did not occur during the time-horizon of interest. Therefore, a binary outcome variable was defined for analysis, indicating whether each event occurred within 1-year of discharge.

### 2.5. Statistical Analysis

Continuous variables are presented as mean and standard deviation, whereas categorical variables are counts and percentages (%). Statistical tests were performed to test for differences between the outcome groups for the primary and secondary outcomes. *p*-values were considered statistically significant at the 0.05 level. To examine the discriminatory ability of the LHFRS stratification, Kaplan–Meier curves were estimated, and log-rank tests were performed to assess differences in survival curves. The Akaike information criterion (AIC) was used to estimate the likelihood of the model to predict/estimate the future values. Finally, the LHFRS score was directly used in the calculation of the concordance probability (C-index, area under the curve [AUC]). The R statistical software (R Core Team (2018). R: A language and environment for statistical computing. R Foundation for Statistical Computing, Vienna, Austria) were used for data analysis. This study complies with the Declaration of Helsinki, and an Institutional Review Board (IRB) approval was obtained.

## 3. Results

Of the 9207 patients in the analysis, 49.5% were male, 67.9% were Caucasian, and 28.4% were African-American. Table 2 displays the univariate summaries of each risk-factor and each demographic attribute at the admission level. All *p*-values appear to show statistically significant differences among the levels of each outcome.

**Figure 1:**
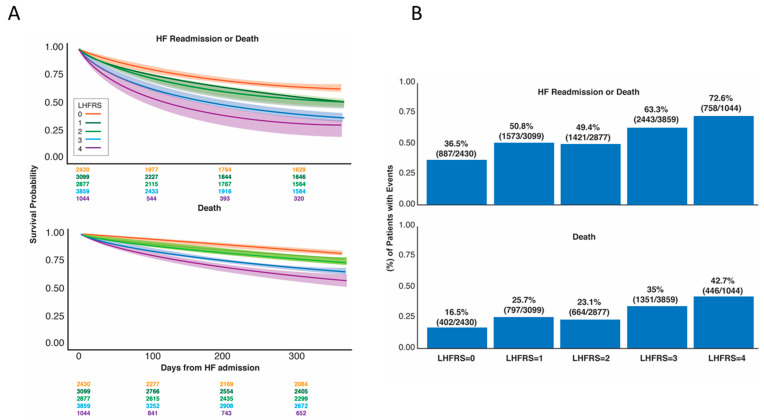
(**A**) Kaplan–Meier curves for the primary and secondary outcomes stratified by the computed Larissa Heart Failure Risk Score (LHFRS); (**B**) Percent of patients experiencing each outcome stratified by the Larissa Heart Failure Risk Score (LHFRS) classification.

Figure 1A shows the Kaplan–Meier curves with 95% confidence bands for the primary and secondary outcomes stratified by the computed LHFRS. Empirically, the curves overall appear to be well-separated and in a fairly consistent order. The log-rank test for both outcomes produced a *p*-value < 0.001.

Figure 1B reiterates the discriminatory ability of the LHFRS by showing the proportion of observed admissions resulting in primary/secondary outcomes at 1-year. It appears the overall trend indicates a consistent relationship between the LHFRS and the risk of the outcome(s). The bootstrapped-validated AUC for the logistic regression model for each outcome using restricted cubic splines for RDW revealed a C-index of 0.64 both for the primary and secondary outcomes, respectively.

**Figure 2:**
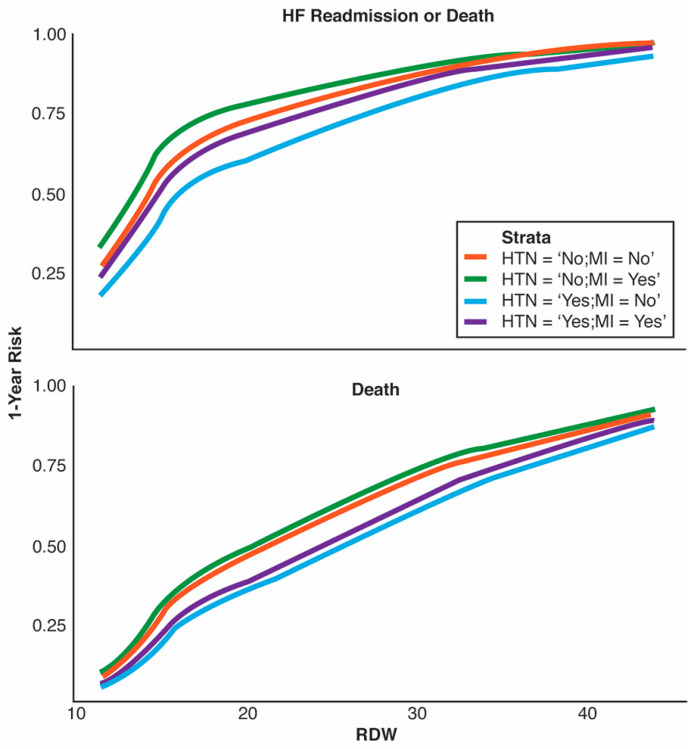
Three-knot cubic spline of red blood cell distribution width (RDW) stratified by hypertension (HTN) and myocardial infarction (MI) groups for the primary and secondary outcomes.

Figure 2 shows the non-linear effect found by the model on each outcome, stratified by combinations of HTN and MI groups. The effects are consistent with the original paper [11] in that patients with a history of MI and no history of HTN are at the highest risk for either event.

**Figure 3:**
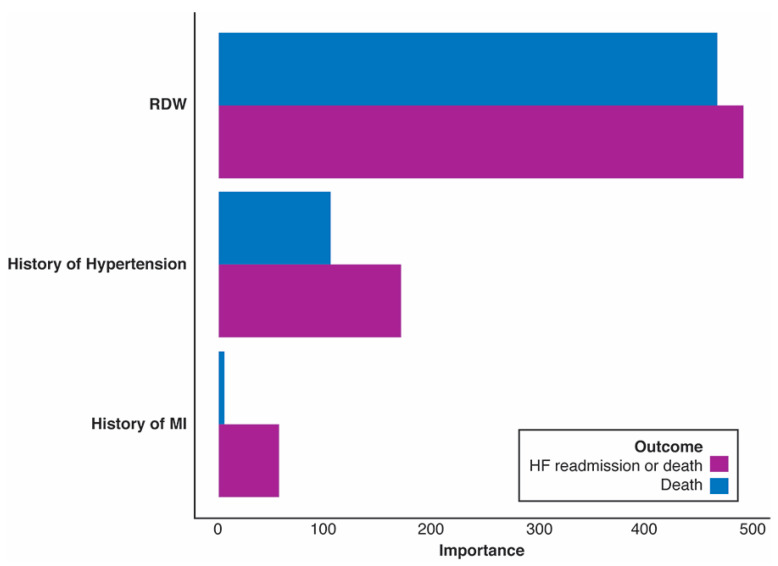
Relative variable importance rankings on the primary and secondary outcomes measured by the increase in Akaike information criterion (AIC) upon removal of each variable from the full model.

We also examined the importance of RDW, HTN, and MI on the primary and secondary outcomes. Logistic regression models were built while modeling the effect of RDW with a three-knot restricted cubic spline. For both outcomes, all variables were found to be statistically significant, including the non-linear effect of RDW on each outcome. The importance of each variable was then ranked by the amount of increase in AIC upon the removal of each variable from the full model. Figure 3 shows the ranked variable importance for each outcome. In both outcomes, RDW was by far the most influential variable in predicting the outcome, followed by the history of HTN and the history of MI. Since all ‘Importance’ measures are positive, this indicates that all variables are influential in explaining the outcomes.

The next step was to investigate the sensitivity and specificity of the LHFRS using a less granular threshold (≤2 versus >2). LHFRS values above 2 were accompanied by low sensitivity and high specificity, as depicted in Table 3.

## 4. Discussion

The present analysis was an external validation of the discriminatory ability of LHFRS in a large population of patients with HHF. Although the performance of LHFRS for the primary endpoint (HF rehospitalizations and/or all-cause mortality) and for the secondary endpoint (all-cause mortality) within 1 year from the initial hospitalization was found to be moderate, the model can be used as a reliable tool for the early detection of high-risk patients as those with a high LHFRS (i.e., 4, *n* = 1044) exhibited significantly worse outcomes in comparison to those with a low LHFRS (i.e., 0, *n* = 2430).

This score consists of three variables (RDW, HTN history, and MI history) that have established prognostic value. RDW is a simple parameter of the complete blood count test (CBC) that expresses the variability of the size of red blood cells (RBCs) and has been traditionally used for the classification of different types of anemia [13]. However, over the last few years, there has been evidence that increased RDW is associated with a poor prognosis in patients with coronary artery disease, stroke, diabetes, and HHF [14]. In our study, patients with increased RDW (≥15%) manifested significantly higher event rates compared with those with RDW values < 15%. Interestingly, as shown in Figure 2, a further increase in RDW beyond 15% is also associated with an incremental risk of adverse events.

Low blood pressure is a negative risk factor, whereas HTN may serve as a protective mechanism in HHF patients, contrary to what is observed in the normal population [15]. The concept of “reactive hypertension”, which proposes the occurrence of a functional cardiac reserve during acute physiologic stress, may be a logical explanation. Patients with impaired functional cardiac reserve and lower blood pressure, even in acute situations (i.e., admission for HF), demonstrate a worse prognosis in comparison to those with higher values of blood pressure [16]. Gheorghiade et al. reported that low blood pressure (<120 mm Hg) at admission was an independent risk factor for an unfavorable prognosis in the participants of the OPTIMIZE-HF registry [17]. In our study, patients with no history of HTN were at highest risk for 1-year adverse outcomes.

The history of MI is also a risk factor for a poor prognosis [18]. The strong association between HF and MI was demonstrated in the Valsartan in Acute Myocardial Infarction Trial (VALIANT) registry, which reported a denovo HF rate of 10.3% in 11,040 stable MI patients during the median 25-month follow-up [19,20]. Regarding our analysis, patients with a history of MI exhibited a higher risk for the primary and secondary endpoints in comparison to those without a history of MI. However, MI history was found to be the least influential variable of LHFRS in predicting the outcomes. This may be attributed to the Cleveland Clinic's successful quality of care performance, as depicted by the low mortality rates among patients who had percutaneous coronary intervention (PCI) procedures and/or presented with acute MI, the short door-to-balloon time, and the 100% administration of all guideline-recommended categories of medical therapy before and after PCI [21]. Thus, it is possible that the performance of LHFRS was “underestimated” in this patient population with unique characteristics and quite different from the central Greece patient cohort used to derive the LHFRS. Furthermore, the modest performance of the LHFRS can partially be explained by the fact that ~45% of patients have an LHFRS equal to 1 or 2; as shown in Figure 1B, these risk groups are nearly indistinguishable.

In the current study, a history of HTN was observed in 46.6% of patients, which is higher than that found in the ADHF/NT-proBNP score (42.9%) [22], comparable to that reported in the ESCAPE study (47%) [23] and ELAN-SCORE (51%) [24], and lower than that presented in the ESC-HF-LT registry (65.6%) [4] and the OPTIMIZE-HF score (72%) [25]. Moreover, in our study, the percentage of MI history was 20.6%, similar to that observed in the OPTIMIZE-HF study (22%) [25] and lower compared to the EFFECT study (37.9%) [26] and the ESCAPE study (44.4%) [23]. The baseline characteristics of the population included in the current study are different from the population examined in our previous report, as far as the race and the percentage of HTN history [11]. Interestingly, our study primary (all-cause mortality and/or HF rehospitalization) and secondary (all-cause mortality) endpoints were found to be higher and similar compared to the ESC-HF-LT registry (53.2% vs. 36% and 27.5% vs. 23.6%, respectively) [4]. Both study endpoints are generally considered robust and well accepted [27].

An interesting review and meta-analysis by Ouwerkerk et al. [28] showed that the mean C-statistic of the risk prediction models in HF was 0.66 ± 0.0005 in general, 0.63 ± 0.001, and 0.71 ± 0.001 with respect to the endpoints of mortality or rehospitalization (combined) and mortality (independently), respectively, which is similar to the current analysis of the LHFRS performance. An external validation of the SENIORS elderly HF risk model at the RICA registry revealed C-statistics of 0.60 and 0.66 for the primary (all-cause mortality or cardiovascular hospitalization) and secondary (all-cause mortality) endpoints, respectively [29]. The validation of the EFFECT score and of the more recent AHEAD Score exhibited a C-index of 0.76 and 0.631 for the endpoint of 1-year all-cause mortality, respectively [26,30].

**Figure 4:**
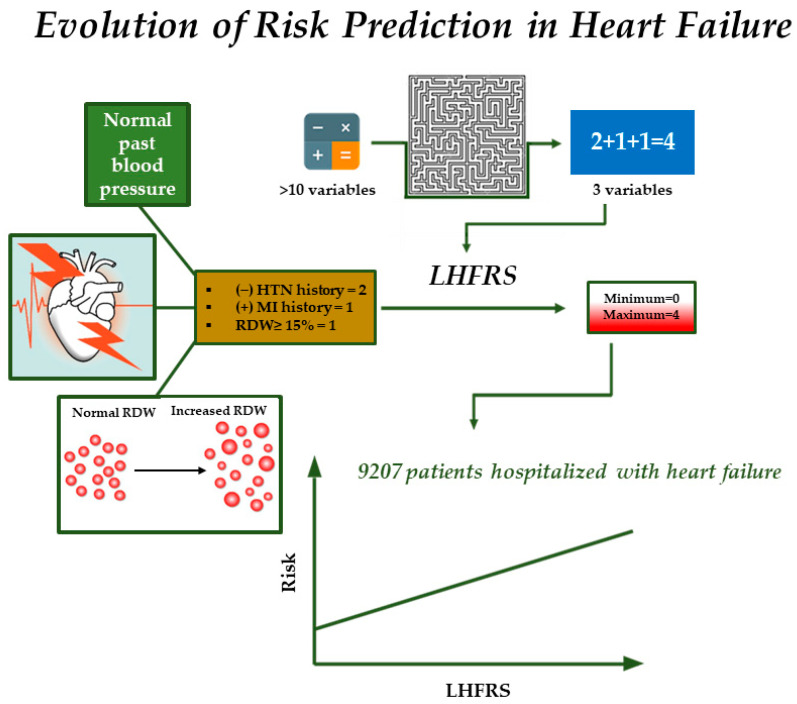
Most prognostic risk models consist of several variables and are complex. The LHFRS is the most parsimonious risk model in HF and includes only three variables (i.e., hypertension history, myocardial infarction history, and red cell distribution width). Its estimation is very simple and does not require the use of sophisticated calculators. The increase in the LHFRS (i.e., from 0 to 4) is related to the incremental rise of unfavorable event risk. Abbreviations: LHFRS, Larissa heart failure risk score; HTN, hypertension; MI, myocardial infarction; RDW, red blood cell distribution width. Adapted with permission from Kitai T. et al. (2020), Copyright © 2023 Elsevier B.V., Ref. [35].

The role of risk scores in HHF is of substantial value as they can be utilized as prognostic tools and assist in clinical decision-making, especially in conditions where the decision must be taken promptly and accurately. However, the majority of the risk scores consist of multiple variables and require the utilization of sophisticated calculators, making their adoption challenging in daily clinical practice [10]. For example, the risk models derived from the OPTIMIZE-HF study, the EFFECT study, and the ESCAPE study include 12, 10, and 8 variables, respectively [23,25,26]. Our score is comprised of just three variables, namely two questions from the patient’s medical history and one marker from the CBC. All of these parameters are easily obtainable, widely available, and low-cost. Therefore, the LHFRS can be practically implemented even by the emergency department. Moreover, a persistent association between the LHFRS and the risk of adverse events was reported in the present analysis, and score values > 2 demonstrated a good specificity for 1-year adverse outcomes. Hence, the utilization of LHFRS may facilitate clinical decision-making (i.e., more aggressive decongestive strategies in “very high-risk patients” with LHFRS ≥3), triage care plans (i.e., admission to the Intensive Care Unit) and close follow-up. Furthermore, a distinct characteristic of LHFRS is its potential to discriminate between the long-term risks of patients with HHF. Typically, HHF prognostic models are developed to predict short-term outcomes [6,31] based on the fact that outcomes during hospitalization or up to 6 months after discharge may seem more relevant to acute pathologies such as HHF syndromes. Nevertheless, long-term outcomes are clinically relevant (i.e., the Seattle HF Score) [32]. For instance, the administration of inotropes in an acute setting may have desirable short-term but neutral or even harmful long-term effects [33,34]. Taken all together, LHFRS exhibited an acceptable discriminatory ability in the present work, despite the fact that it is the most parsimonious long-term risk model in HHF (Figure 4) [35].

### 4.1. Limitations

This study has several limitations that need to be addressed. First, it is a retrospective study, and hence the results depend on the accuracy of the data collection. Second, the single-center nature of the present work in a high-volume tertiary center may limit its generalizability in diverse clinical settings. This is especially true regarding the predictive value of one of the constituents of the LHFRS, namely the MI history. However, most likely, MI treatment is less efficient in “real-world” centers, increasing the predictive value of the MI history and consequently of the LHFRS in this setting. Thirdly, patients with Hg values < 10 g/dL were excluded, and, therefore, this score was not tested in severely anemic patients. However, anemia, which may influence RDW values, was present in only 18% of the HHF patients in Get With the Guidelines—Heart Failure [36]. The prognosis determined at admission may be affected by several post-discharge factors, including medical interventions, revascularization, cardiac resynchronization therapy, and implantable cardioverter defibrillator implantation. Assigning medical history by manual chart review is timely but accurate. Assigning medical history from electronic databases are challenging and dependent upon discrete searchable variables or complex algorithms to extract specific medical histories. Various algorithms were utilized to develop methods to determine medical history, including HTN and MI. Lastly, a number of predictive models have been developed for HF patients based on artificial intelligence over the last few years [37,38,39]. However, these models have important limitations, such as the inability to account for time-to-event variations as well as the “interpretability issue” (i.e., poor explanation of the predictions) [40].

### 4.2. Future Perspectives

The LHFRS has demonstrated the ability to predict risk in hospitalized HF patients for subsequent events up to 1 year post-discharge, irrespective of the ejection fraction. An elevated LHFRS at the time of HF hospitalization might prospectively identify patients with advanced HF, a population with an increased risk of adverse outcomes [41]. Long-term outcomes (1 year post-discharge) may be clinically important, especially with respect to identifying advanced HF patients that are potential candidates for left ventricular assist devices (LVAD) and/or heart transplantation. Furthermore, the combination of the LHFRS with novel markers of prognosis in HF, such as spot urinary sodium, may be a fruitful area of research [42].

## 5. Conclusions

LHFRS is easily calculated and can be utilized as a basis for risk prediction in patients with HHF. An elevated LHFRS of ≥3 is associated with a 1-year mortality rate over 35%. Further studies investigating the role of this simple prognostic tool in HF patients are needed.

## Figures and Tables

**Table 1:** ICD codes and descriptions of the variables used in the analysis.

**HEART FAILURE**		
ICD9	ICD10	Description
I11.0		Hypertensive heart disease with heart failure
I11.0		Hypertensive heart failure
I13.0		Hypertensive heart and chronic kidney disease with heart failure and stage 1 through stage 4 chronic kidney disease, or unspecified chronic kidney disease
I13.2		Hypertensive heart and chronic kidney disease with heart failure and stage 5 chronic kidney disease, or end-stage renal disease
I50.1		Cardiac asthma
I50.1		Edema of the lung with heart disease NOS
I50.1		Edema of the lung with heart failure
I50.2		Systolic-congestive heart failure
I50.21		Acute systolic congestive heart failure
I50.3		Diastolic congestive heart failure
I50.31		Acute diastolic congestive heart failure
I50.4		Combined systolic (congestive) and diastolic (congestive) heart failure
I50.9		Biventricular congestive heart failure
I50.9		Cardiac, heart, or myocardial failure (NOS)
I50.9		Right ventricular failure (secondary to left heart failure)
398.91	I09.81	Congestive rheumatic heart failure
402.01		Malignant hypertensive heart disease with heart failure
402.11		Benign hypertensive heart disease with heart failure
402.91		Unspecified hypertensive heart disease with heart failure
404.01		Hypertensive heart and chronic kidney disease, malignant, with heart failure and chronic kidney disease stage I through stage IV, or unspecified
404.03		Hypertensive heart and chronic kidney disease, malignant, with heart failure, and with chronic kidney disease stage V or end-stage renal disease
404.11		Benign hypertensive heart and renal disease with congestive heart failure
404.12		Benign hypertensive heart and renal disease with renal failure
404.13		Benign hypertensive heart and renal disease with congestive heart failure and renal failure
404.91		Hypertensive heart AND chronic kidney disease with congestive heart failure
404.92		Hypertensive heart and renal disease with renal failure
404.93		Hypertensive heart and renal disease with both (congestive) heart failure and renal failure
428.0	I50.9	Congestive heart failure
428.1	I50.1	Left-Sided Heart Failure
428.20	I50.20	Heart Failure, Systolic
428.21		Acute systolic heart failure
428.22	I50.22	Chronic systolic heart failure
428.23	I50.23	Acute on chronic systolic heart failure
428.30	I50.30	Heart Failure, Diastolic
428.31		Acute diastolic heart failure
428.32	I50.32	Chronic diastolic heart failure
428.33	I50.33	Acute on chronic diastolic heart failure
428.40	I50.40	Unspecified combined systolic (congestive) and diastolic (congestive) heart failure
428.41	I50.41	Acute combined systolic and diastolic heart failure
428.42	I50.42	Chronic combined systolic and diastolic heart failure
428.43	I50.43	Acute on chronic combined systolic and diastolic heart failure
428.9	I50.9	Heart failure
**HISTORY OF HYPERTENSION**		
ICD9 Code	ICD10 Code	
997.91	I10-I15	
401.9	I10	
401.1		
**HISTORY OF MYOCARDIAL INFARCTION**		
ICD9	ICD10	Description
410.82		Acute myocardial infarction of other specified sites, subsequent episode of care
410.8		Acute myocardial infarction of other specified sites, episode of care unspecified
410.9	I21.3	Acute myocardial infarction
410.7	I21.4	Acute subendocardial myocardial infarction
410.1		Acute myocardial infarction of the other anterior wall, episode of care unspecified
I21.02		ST elevation (STEMI) myocardial infarction involving the left anterior descending coronary artery
I21.3		ST elevation (STEMI) myocardial infarction of an unspecified site
I21.4		Non-ST elevation (NSTEMI) myocardial infarction
410.92		Acute myocardial infarction, unspecified site, subsequent episode of care
I21.19		ST elevation (STEMI) myocardial infarction involving other coronary arteries of the inferior wall
410.71		Acute myocardial infarction, subendocardial infarction, initial episode of care
410.72		Subendocardial infarction, subsequent episode of care
410.4		Acute myocardial infarction of the other inferior wall, episode of care unspecified
I21.09		ST elevation (STEMI) myocardial infarction involving other coronary arteries of the anterior wall
410.41		Acute myocardial infarction of the other inferior wall, initial episode of care
410.51		Acute myocardial infarction of the other lateral wall, initial episode of care
410.01		Acute myocardial infarction of the anterolateral wall, initial episode of care
410.6		True posterior wall infarction, episode of care unspecified
I21.29		ST elevation (STEMI) myocardial infarction involving other sites
410.91		Acute myocardial infarction, unspecified site, initial episode of care
410.11		Acute myocardial infarction of the other anterior wall, initial episode of care
410.21		Acute myocardial infarction of the inferolateral wall, initial episode of care
410.31		Acute myocardial infarction of the inferoposterior wall, initial episode of care
410.81		Acute myocardial infarction of the other specified sites, initial episode of care
I21.11		ST elevation (STEMI) myocardial infarction involving the right coronary artery
I22.2		Subsequent non-ST elevation (NSTEMI) myocardial infarction
410.61		True posterior wall infarction, initial episode of care
410.12		Acute myocardial infarction of the other anterior wall, subsequent episode of care
410.32		Acute myocardial infarction of the inferoposterior wall, subsequent episode of care
410.42		Acute myocardial infarction of the other inferior wall, subsequent episode of care
410.02		Acute myocardial infarction of the anterolateral wall, subsequent episode of care
410.52		Acute myocardial infarction of the other lateral wall, subsequent episode of care
410.62		True posterior wall infarction, subsequent episode of care
410.5		Acute myocardial infarction of the other lateral wall, episode of care unspecified
410.7		Acute myocardial infarction, subendocardial infarction
410.5		Acute myocardial infarction of the other lateral wall
410		Acute myocardial infarction of the anterolateral wall
410		Acute myocardial infarction of the anterolateral wall, episode of care unspecified
410.8		Acute myocardial infarction at other specified sites
I21.01		ST elevation (STEMI) myocardial infarction involving the left main coronary artery
410.2		Acute myocardial infarction of the inferolateral wall, episode of care unspecified
410.3		Acute myocardial infarction of the inferoposterior wall, episode of care unspecified
I22		Myocardial Infarction

**Table 2:** A univariate summary of risk-factors in addition to demographic variables at the admission level. Counts (percentages) were computed for categorical variables and the mean (SD) for continuous ones. For categorical variables, a Chi-square test of independence was performed. For numeric variables, a Wilcoxson rank-sum test was performed. *p*-values testing differences among outcome groups are also displayed.

Variable			Outcome					
			1-Year HF Readmission or Death		*p*-Value	1-Year Death		i-Value
			No	Yes		No	Yes	
Gender								
	Female		3172 (50.9%)	3291 (46.5%)	<0.01	4742 (49.1%)	1721 (47%)	0.03
	Male		3055 (49.1%)	3791 (53.5%)		4907 (50.9%)	1939 (53%)	
Race								
	African-American		1911 (30.7%)	2347 (33.1%)	<0.01	3356 (34.8%)	902 (24.6%)	<0.01
	Caucasian		4067 (65.3%)	4471 (63.1%)		5895 (61.1%)	2643 (72.2%)	
	Other		119 (1.9%)	83 (1.2%)		173 (1.8%)	29 (0.8%)	
	Missing		130 (2.1%)	181 (2.6%)		225 (2.3%)	86 (2.3%)	
Age at admission			72.52 (14.49)	75.4 (14.28)	<0.01	71.91 (14.5)	79.69 (12.7)	<0.01
Hypertension								
		No	2874 (46.2%)	4232 (59.8%)	<0.01	4826 (50%)	2280 (62.3%)	<0.01
		Yes	3353 (53.8%)	2850 (40.2%)		4823 (50%)	1380 (37.7%)	
Myocardial infarction								
		No	5154 (82.8%)	5414 (76.4%)	<0.01	7745 (80.3%)	2823 (77.1%)	<0.01
		Yes	1073 (17.2%)	1668 (23.6%)		1904 (19.7%)	837 (22.9%)	
* RDW (%)								
		<15	3218 (51.7%)	2438 (34.4%)	<0.01	4561 (47.3%)	1095 (29.9%)	<0.01
		>=15	3009 (48.3%)	4644 (65.6%)		5088 (52.7%)	2565 (70.1%)	

* RDW: red blood cell distribution width.

**Table 3:** Examining the sensitivity/specificity of using a less granular threshold of the LHFRS (<=2 vs. >2) for: (a) all-cause death or rehospitalization for heart failure; (b) all-cause death.

		Death or HF Re-Hospitalisation	
		Yes	No
LHFRS	>2	3201	1702
	≤2	3881	4525
Sensitivity [(95% CI)] = 0.452 (0.44, 0.464), Specificity [(95% CI)] = 0.727 (0.716, 0.738)			
		Death	
		Yes	No
LHFRS	>2	1797	3106
	≤2	1863	6543
Sensitivity [(95% CI)] = 0.491 (0.475, 0.507), Specificity [(95% CI)] = 0.678 (0.669, 0.687)			

## Data Availability

All data generated or analyzed during this study are included in this published article.
